# Axonal Transport and Neurodegeneration: How Marine Drugs Can Be Used for the Development of Therapeutics

**DOI:** 10.3390/md14050102

**Published:** 2016-05-19

**Authors:** Joseph A. White, Rupkatha Banerjee, Shermali Gunawardena

**Affiliations:** Department of Biological Sciences, The State University of New York at Buffalo, Buffalo, NY 14260, USA; jwhite2@buffalo.edu (J.A.W.); rupkatha@buffalo.edu (R.B.)

**Keywords:** axonal transport, molecular motor proteins, neurodegenerative diseases

## Abstract

Unlike virtually any other cells in the human body, neurons are tasked with the unique problem of transporting important factors from sites of synthesis at the cell bodies, across enormous distances, along narrow-caliber projections, to distally located nerve terminals in order to maintain cell viability. As a result, axonal transport is a highly regulated process whereby necessary cargoes of all types are packaged and shipped from one end of the neuron to the other. Interruptions in this finely tuned transport have been linked to many neurodegenerative disorders including Alzheimer’s (AD), Huntington’s disease (HD), and amyotrophic lateral sclerosis (ALS) suggesting that this pathway is likely perturbed early in disease progression. Therefore, developing therapeutics targeted at modifying transport defects could potentially avert disease progression. In this review, we examine a variety of potential compounds identified from marine aquatic species that affect the axonal transport pathway. These compounds have been shown to function in microtubule (MT) assembly and maintenance, motor protein control, and in the regulation of protein degradation pathways, such as the autophagy-lysosome processes, which are defective in many degenerative diseases. Therefore, marine compounds have great potential in developing effective treatment strategies aimed at early defects which, over time, will restore transport and prevent cell death.

## 1. Introduction

**Figure 1:**
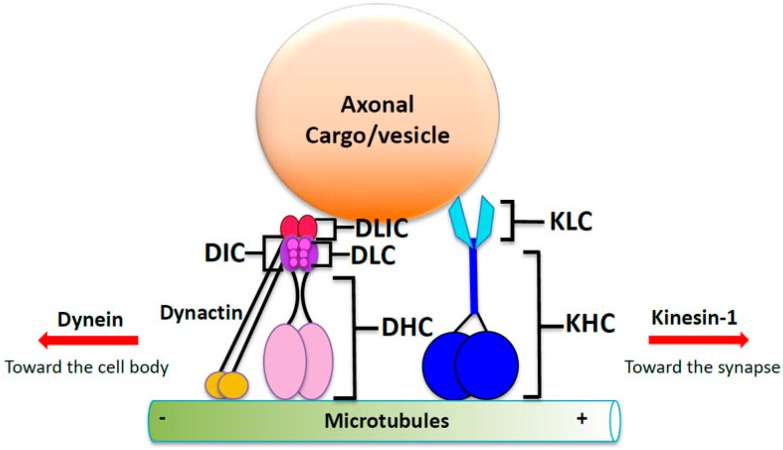
Axonal transport and a hypothetical cargo-motor complex. Schematic diagram of a cargo-motor complex undergoing motility on a microtubule (MT). The anterograde motor kinesin-1 is composed of two subunits. Kinesin heavy chain (KHC) contains the motor domain, which binds MTs and possesses ATPase activity. Kinesin moves cargo from the cell body to the nerve terminal or synapse. Kinesin light chain (KLC) links kinesin to the cargo or vesicle. The retrograde motor dynein is also composed of several subunits. The dynein heavy chain (DHC) contains the motor domain. Dynein light chains (DLCs), dynein intermediate chains (DICS) and dynein light-intermediate chains (DLICs) aid in attaching the cargo or vesicle to the motor for transport toward the cell body. Dynactin is a multi-subunit protein that includes p150glued. The p150glued subunit attaches to MTs and is thought to be important for the regulation of dynein. Other subunits of dynactin aid in the attachment of cargo.

Axonal transport refers to the long-range transport of components within narrow-caliber axons that can extend more than a meter in humans. In axons, cargoes are moved bi-directionally on polarized microtubule (MT) tracks; from the cell body or soma to the nerve terminal where the synapse is located (Figure 1). Transport of cargoes from the cell body toward the synapse is termed anterograde transport while transport from the synapse to the cell body is termed retrograde transport. This directionality is, in part, due to the inherent polarity of the microtubules which contain plus (+) and minus (−) ends. The molecular motor proteins kinesin and dynein move using the polarity of the MTs, which dictate the directionality of cargo movement (Figure 1). Kinesins are responsible for transporting cargoes essential for synaptic function in the anterograde direction while dynein moves cargoes that need to be brought back to the cell body from the distal synapse in the retrograde direction. Molecular motors are protein complexes which, when bound to MTs, “walk” up or down the MT. Molecular motor proteins carry a variety of different cargo types including synaptic vesicles, organelles, and even RNA for fast transport within the axon on MTs.

Axonal transport defects are proposed to instigate degeneration, or axonopathy, leading to neuronal cell death in several neurodegenerative diseases such as Alzheimer’s disease (AD) [1,2,3], Huntington’s disease (HD) [4,5], and Amyotrophic Lateral Sclerosis (ALS) [6]. Although the exact mechanism of how axonal degeneration occurs is not completely understood, several factors pertaining to axonal transport seem to be potential progenitors of degeneration. Destabilization and fragmentation of MTs as seen in tauopathies [7,8], loss of function of molecular motors or deregulation of motor activity [3,5,9,10,11], and aggregation of proteins due to deficits in cellular clearance pathways (the autophagy-lysosome pathway and the ubiquitin-proteasome pathway [12]), all of which disrupt transport, and have been associated with axonal degeneration in many neurodegenerative disorders. MT maintenance and motor protein regulation are essential for proper transport within axons [11,13], and for the maturation of autophagosomes within growing axons [14]. Work has shown that malfunctions in these processes can disrupt long-distance transport which, in turn, can cause axonal degeneration, suggesting that the disruption of the transport pathway is likely an early event in disease progression [15,16,17].

Intriguingly, work has shown that mutations in molecular motors can cause neurodegeneration. For example, mutations in the kinesin subunit KIF1B cause Charcot-Marie-Tooth syndrome type 2A (CMT2A) [18]. Hereditary spastic paraplegia (HSPs) is caused by mutations in the kinesin-1 heavy chain subunit KIF5A [19]. Mutations in dynein heavy chain (DYNHC1) also cause a form of CMT type O (CMTO) [20]. Dynein mutations were also found to cause motor-neuron diseases including ALS [19], and a form of l-Dopa-resistant PD called Perry syndrome [21]. In addition, a number of proteins, in which mutations cause common neurodegenerative diseases such as AD and HD, have also been shown to either be transported within axons or have important roles in the axonal transport pathway. The amyloid precursor protein (APP), which undergoes cleavage to form amyloid-beta, and is aggregated in senile plaques in AD, is transported within axons via the molecular motor kinesin-1 [2,22,23,24]. Expression of familial AD (fAD) forms of APP result in the formation of axonal blocks which are correlated with neuronal cell death [2], and axonal swellings are seen early in AD patient brains before the onset of neuropathology [1]. The HD protein huntingtin (HTT) is also proposed to move essential cargo complexes during axonal transport [25,26,27,28]. Recently, HTT has been shown to influence the axonal transport of a subset of Rab GTPases [28], brain-derived neurotrophic factor (BDNF) [27], and components of the autophagy pathway [29]. Expansion of polyQ repeats in the context of HTT perturbs transport [5,30] and causes cytoplasmic accumulations within axonal projections early; before the onset of behavioral defects in both mice and HD patients [4]. Early axonal transport deficits have also been found in PD models [31] and human PD brains show decreased levels of kinesin and dynein at early stages of PD [32]. In addition, defects in the transport of neurofilaments have also been seen early, before behavioral defects in mouse models of Amyotrophic Lateral Sclerosis (ALS) [33]. Therefore, targeting therapeutics to modulate axonal transport defects could help restore normal functionality; eliminating initiation of deleterious pathways during the progression of neurodegenerative diseases.

Increased exploration of the world’s oceans and research on marine organisms in recent years has led to a tremendous interest in the identification of naturally occurring compounds produced by living organisms [34]. These natural products are continuing to give rise to potentially viable therapeutic compounds [35,36]. In this review, we examine a number of marine-derived drugs, discuss potential mechanisms of how they affect the axonal transport pathway, and evaluate how these compounds can be used to potentially develop effective treatment strategies for axonal transport defects observed in many neurodegenerative diseases.

## 2. Microtubules: The Tracks of the Axonal Highway

**Figure 2:**
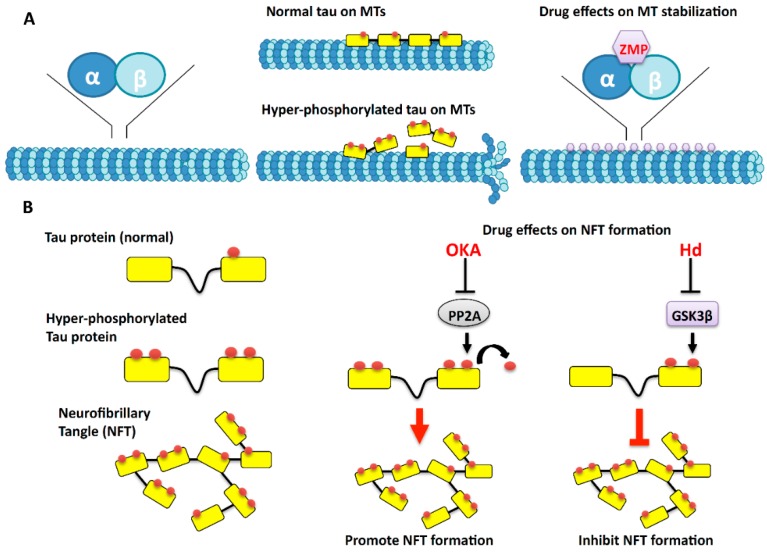
Proposed effects of marine drugs on microtubule stabilization. (**A**) Microtubules (MTs) are polarized structures that are composed of filaments containing alpha and beta tubulin subunits (left). Tau, an MT-associated protein (MAP), stabilizes MT tracks within axons by binding to tubulin. Tau is normally phosphorylated and in disease states is hyper-phosphorylated causing destabilization of MTs (middle). Zampanolide (ZMP) is a drug derived from the marine sponge *Fasicospongia rimosa* interacts with tubulin and helps to stabilize MTs; (**B**) Hyper-phosporylation of tau causes tau to aggregate and form neurofibrillary tangles (NFTs) which are seen in many disease states (left). Okadaic acid (OKA) inhibits protein phosphatase 2A, which increases the level of phosphorylated tau (middle). Hymenaldisine (Hd) inhibits the kinase activity of glycogen synthase kinase 3-beta (GSK3β) and decreases the level of phosphorylated tau. This drug could potentially inhibit the formation of NFTs (right) by inhibiting tau phosphorylation.

Microtubules (MTs) are one of the major cytoskeletal components of neurons and extend along the length of the axon. MTs are highly dynamic polymers that grow and shrink at rapid rates. MTs are composed of alternating α- and β-tubulin and each α- or β-monomer has a binding site for a single molecule of GTP, which regulates polymerization [37,38] (Figure 2A). Tubulin dimers polymerize to form MTs, consisting of 13 linear proto-filaments assembled around a hollow core [39]. Since the proto-filaments are composed of head-to-tail arrays of tubulin dimers, MTs contain two distinct ends: a fast-growing plus-end and a slow-growing minus-end [40]. In the stable state, MTs are predominately composed of GDP-bound β-tubulin proto-filaments. In the dynamic state, which is called “tread milling”, GDP-bound tubulin is continually lost from the minus-end and is replaced by the addition of GTP-bound tubulin at the plus-end [41]. In MTs, GTP hydrolysis also results in dynamic instability, where individual MTs alternate between cycles of growth and shrinkage [42].

Axons are long cable-like projections containing MT tracks. Many organelles and proteins synthesized at the cell body are transported down the axon along MTs. Therefore MTs serve as the tracks on which motor proteins transport organelles and other vesicular cargoes in axons. Loss of MTs within axons is associated with a large number of neurodegenerative diseases including Alzheimer’s disease (AD) [43]. MT loss is also well demonstrated in a class of diseases known as tauopathies or frontotemporal dementia (FTD). In these diseases, the tau protein is hyper-phosphorylated causing destabilization of MTs [44,45] (Figure 2B). Tau is a microtubule-associated protein (MAP) that stabilizes MTs by promoting MT nucleation and elongation while preventing disassembly. However, hyper-phosphorylation of tau promotes aggregation of tau and reduces the ability of tau to bind to MTs. As a result, MTs become destabilized. Hyper-phosphorylated tau aggregates into paired helical filaments (PHFs) and straight filaments (SFs) that result in the formation of neurofibrillary tangles (NFTs) [46,47]. Hyper-phosphorylated tau is the main component of NFTs found in the brains of patients with AD [48,49] and FTD [50]. Recent studies have indicated that reduced activity of phosphatases like protein phosphatase 2A (PP2A) can lead to hyper-phosphorylation of tau [49]. In neurons, PP2A is associated with the activation of major kinases including MT-associated protein kinase (MAPK), extracellular signal-regulated kinase (ERK), c-jun *N*-terminal kinase (JNK), protein kinases C and A (PKC and PKA), and glycogen synthase kinase 3 beta (GSK-3β). Increased activities of these kinases are also proposed to promote hyper-phosphorylation of tau via unknown mechanisms [49]. Additionally, oxidative stress and mitochondrial abnormalities have also been reported to cause tau hyper-phosphorylation [47].

## 3. The Effects of Marine Drugs on Microtubules

One mechanism by which drugs affecting MT stabilization could act to alleviate disease conditions is to promote the destabilization of MTs and reduce impairment in the trafficking of vital cargoes. Such drugs may also stabilize MTs in a manner that allows for MTs to be repaired more efficiently. Studies show that the MT-stabilizing drug taxol can augment regeneration of optic nerves following injury [51]. Additionally, taxol facilitates the regeneration of injured spinal cords [52]. Such observations provide evidence that drugs which stabilize MTs can act to promote axon regeneration. Proper axonal transport along MT tracks ensuring ordered transport of organelles and vesicles to sites of injury could be one strategy that might enhance the capacity of the injured axons to regenerate.

Bioactive compounds from marine organisms have been found to affect MT dynamics by affecting MT polymerization, assembly, and disassembly. One such compound, Zampanolide (ZMP), was reported in the marine sponge, *Fasicospongia rimosa* [53]. Another ZMP isolated from another marine sponge *Cancospongia mycofijiensis* was found to enhance tubulin assembly in the absence of MT-associated proteins (MAPs) [54]. Therefore, ZMP may act as an MT-stabilizing agent (MSA). It was shown that ZMP interacts covalently with residues N228 and H229 of un-polymerized tubulin (Figure 2A). Although tubulin is covalently modified on ZMP binding, the morphology of the MTs assembled in the presence of ZMP is not altered. It was proposed that the allosteric interaction of ZMP with the M-loop of β-tubulin leads to the stabilization of MTs. Interestingly, Dactylolide (DAC), another marine drug isolated from *Dactylospongia* sp. is structurally similar to ZMP [55]. DAC has similar binding sites as ZMP, but is less potent than ZMP as an MSA [54] showing reduced binding kinetics with MTs compared to ZMP.

The MT-stabilizing protein, tau, is also affected by the marine drug Hymenialdisine (Hd). Hd is derived from various marine sponges including *Hymeniacidon aldis*, *Axinella verrucosa* and *Acanthella aurantiaca* [56]. *In vivo* studies in cerebellar granule cells revealed that Hd inhibits phosphorylation of tau at sites that are hyper-phosphorylated by GSK-3β and cyclin-dependent kinase 5 (CDK5). It does so by competing with ATP for binding to these kinases [57]. In addition, Hd also caused extensive branching and spreading of axons *in vivo* [57]. These phenotypes were accompanied by shortening of axon length in areas characterized by loss of stable MTs. It is proposed that these phenotypes arise due to the role of Hd inhibiting GSK-3β-dependent phosphorylation of MAP-1B, which acts to stabilize MTs, and is present along the entire length of the axon (Figure 2B). Hd can also induce loss of MAP-1B, which correlates with the loss of stable MTs resulting in shortening of axons [57].

A secondary metabolite, Peloruside A isolated from the sponge *Mycale hentscheli* [58] is similar to ZMP in that it can act as a microtubule-stabilizing agent [59]. It is proposed that Peloruside A stabilizes MTs by acetylating tubulin and promoting interactions across the tubulin inter-dimer interface. Discodermolide, extracted from the deep-sea sponge *Discoderma dissolute*, prevented cell proliferation [60]. Electron microscopy data revealed that Discodermolide can stabilize MTs by targeting an additional binding site to the pore of the MT, distinct from the binding site at the lumen of the MTs. Binding to this site is the first ligand-protein interaction that precedes binding to the luminal site of MTs [61]. This additional binding site may be the reason why Discodermolide remains the most potent natural MT-stabilizing drug currently discovered [62].

While stabilizing MTs can have positive effects on axonal transport, loss of MTs by treatment with microtubule destabilizing agents can lead to negative effects on axonal transport. Drugs that destabilize MTs can potentially allow us to better understand how loss of MT stabilization affects axonal transport and leads to neurodegenerative disease. Furthermore, application of these drugs could serve as simple models of disease where we can study the implications of MT destabilization. Several metabolites from marine organisms have been implicated in destabilizing MTs and are discussed below. Okadaic acid (OKA), a drug from the marine sponge *Halichondria okadai*, selectively inhibits protein phosphatases PP1 and PP2A. OKA was found to increase levels of phosphorylated tau (Figure 2B) and GSK-3β by blocking PP2A activity [63], causing the accumulation of NFTs, resulting in AD-like neuropathology [49]. OKA destabilizes MTs, via tau phosphorylation and inhibits neurite outgrowth in neuronal cultures [64]. However, this effect of OKA was reversed by treating the neurons with previously described MT-stabilizing agent Peloruside A. Therefore, it can be proposed that OKA-induced increases in GSK-3β activity results in hyper-phosphorylation of tau, which causes MT destabilization. It should be noted, however, that inhibiting phosphatases such as PP1 and PP2A which have a wide range of targets within a cell could also affect MT stabilization via a GSK3β-independent mechanism. The MT-stabilizing drug Peloruside A on the other hand might exert its effect by decreasing GSK-3β activity; thereby decreasing tau hyper-phosphorylation which may alleviate disease-induced MT destabilization.

Dolastatin 10 is an example of an MT-destabilizing drug that is isolated from the sea hare, *Dolabella auricularia* [65]. This drug inhibits MT assembly by affecting tubulin-dependent GTP hydrolysis [66]. By dampening MT dynamics, Dolastatin and its analogs are thought to be effective in inhibiting cell proliferation *in vitro* [67,68,69]. Dolastatins act by binding to the vinca alkaloid domain of tubulin, a location where vinca alkaloids such as vincistrine bind. Vinca alkaloids are nitrogenous bases that bind to tubulin both *in vivo* and *in vitro* and inhibit the polymerization of tubulin to MTs [69]. In doing so, Dolastatins act as non-competitive inhibitors of vincistrine and other vinca alkaloids [66]. Symplostatin, isolated from *Symploca hydnoides*, has structures very similar to Dolastatin and serves as a potent MT inhibitor [70].

Halichondrin, isolated from *Halichondria okadai*, has a mode of action distinct from Dolastatins [71]. Halichondrin destabilizes MTs by inducing depolymerization of preassembled MTs [72]. E7389 is a synthetic analog of Halichondrin B that functions by an end-poisoning mechanism in which tubulin monomers are sequestered into aggregates. As a result, MT growth is suppressed and no MT shortening is observed since sequestration of un-polymerized tubulin lowers the concentration of free tubulin available for polymerization [73].

Cryptophycin isolated from freshwater cyanobacterium *Nostoc* sp. also suppresses MT dynamics [74]. Like Dolastatin, this drug inhibits nucleotide exchange and causes tubulin to aggregate into ring-shaped oligomers. Cryptophycin interacts with the vinca alkaloid domain and inhibits tubulin’s hydrolyzation of GTP. Although Cryptophycin was found to act in a manner similar to Dolastatins, this drug also mediated its effect by an end-poisoning mechanism similar to halichondrins [74]. Therefore, the different actions of these drugs might provide us with valuable insight into the molecular mechanisms that cause MT loss, thereby providing therapies to counteract MT defects found in many neurodegenerative diseases.

## 4. Molecular Motor Proteins Kinesin and Dynein: The Engines of Microtubule Transport

Transport of essential components along MTs relies on molecular motors kinesin and dynein. Motor proteins move cargo, such as organelles, synaptic proteins, and mRNA, bi-directionally along the axon [15]. Conventional kinesin, also known as kinesin-1, is a plus-end-directed motor protein that is responsible for anterograde transport, motility of cargos from the cell body to distal synapses where these components are then utilized. In contrast, cytoplasmic dynein is the minus-end-directed motor protein that moves cargoes that need to be brought back to the cell body for recycling or degradation during retrograde transport [75].

Kinesin-1 is part of a superfamily of kinesin proteins that carry distinct cargo complexes within axons. Kinesin-1 motor protein, also known as the conventional anterograde motor, is highly conserved [75]. Kinesin-1 is a tetramer composed of two heavy chains (120 kD) and two light chains (62 kD) [76,77] (Figure 1). The kinesin heavy chain (KHC) subunit is the motor domain, which contains sites that bind MTs and ATP. Hydrolysis of ATP provides energy for processive motion along MTs [78]. KHC also contains a stalk domain that is required for dimerization of the heavy chains, and a *C*-terminal tail domain that interacts with the kinesin light chain (KLC) subunit, which enables associations with a variety of cargoes [79,80,81,82]. Some of the cargoes transported by kinesin-1 include SNAP 25, APP, and organelles such as mitochondria and endoplasmic reticulum [83].

Cytoplasmic dynein is a complex motor protein that moves retrogradely within axons. Dynein is composed of multiple subunits consisting of two heavy chains (DHC) that contain the catalytic activity of the motor protein, intermediate chains (DIC), light-intermediate chains (DLICs), and light chains (DLC) [84,85,86,87] (Figure 1). The two heavy chains contain the motor domain and have ATPase activity. Similar to kinesin, dynein’s light and intermediate chains enable dynein to bind a variety of cargoes including β-Catenin [88], neurotrophin receptors [89], and lysosomes [90]. A complex of proteins called dynactin interacts with dynein to mediate retrograde transport (Figure 1). The p150Glued subunit is the largest subunit of the dynactin complex and binds to both MTs and DICs [86,91,92]. Other components of dynactin include dynamitin (p50), p62, actin-interacting protein (Arp) 1 and Arp 11, actin, p24, p25, p27 and capZ α and β [93,94]. These proteins promote the ability of dynein to bind to a variety of vesicles or cargoes such as bicaudal 2 (BICD2) and Rab6-bound organelles [95]. Interestingly, work has also shown that dynactin can play a role in regulating directionality of motility by association with dynein and kinesin via HTT phosphorylation [96].

Since molecular motors are instrumental in the transport of vesicles and organelles within axons, it is not surprising that they are highly regulated. Phosphorylation of motor proteins has been shown to affect their attachment to cargo complexes [97] and MTs [98], and to regulate the directionality of their movement [9]. Recent *in vivo* and *in vitro* evidence for a variety of cargo/vesicles suggests that a single vesicle is likely to be simultaneously bound by many different motors as indicated by their bi-directional motility [5,9,28]; not only by conventional kinesin and dynein but also different types of kinesins such as kinesin-3 [99]. Therefore, binding of kinesin and dynein to vesicles is likely to be highly coordinated for the proper movement of cargo bi-directionally within axons. Indeed, at least two models have been proposed to define the bi-directional motility observed both *in vivo* and *in vitro.* The Tug-of-War model suggests that there is a constant battle between the two opposing motors and the motor that wins defines directionality of cargo movement [100]. The coordination model suggests that bi-directional movement is much more regulated and that the activities of both kinesin and dynein motors are dependent on the motor activity of each motor because loss of one type of motor affects motility in both directions [11,13,101,102]. Additionally, attachment of motors to MTs appears to be highly regulated by various kinases that affect cargo velocity and direction of movement. For example, protein kinase B (PKB/Akt) can alter the direction of huntingtin-containing vesicles via phosphorylation of the huntingtin protein (HTT) [96]. Kinases can also directly affect the binding of motors to vesicles/cargoes themselves. Recent work in *Drosophila* showed that GSK3β over-activity not only cause axonal blocks, but also increase binding of both kinesin and dynein motors to membranes, thus suggesting that GSK3β activity is important for motor protein binding to cargo or vesicles [9]. Work *in vitro* has shown that GSK3β activity can also release motors from vesicles by the phosphorylation of KLC [97]. Intriguingly, the neurodegenerative disease, human spastic paraplegia, is caused by mutations in KHC, the motor subunit of kinesin-1 [19,103], resulting in reduced anterograde transport, synaptic defects, and posterior paralysis [104]. Perhaps drugs that promote anterograde transport could alleviate or delay the onset of such diseases. Below we discuss a number of marine organism-derived drugs that affect kinases that have the potential to work as regulators of molecular motors. Such candidate drugs could alleviate molecular motor-dependent defects; preventing perturbation of axonal transport motors and degeneration.

## 5. The Effects of Marine Drugs on Molecular Motors

Proper transport of cargo within long, narrow caliber axons are essential for cell viability. Several studies have shown that mutations in motor proteins cause neurodegenerative diseases. Mutations in the motor domain of KIF5A, one of the KHC subunits has been shown to cause hereditary spastic paraplegia (HSP) [19]. Charcot-Marie-Tooth type 2 patients have also been found to have a mutation in the stalk domain of KIF5A [103]. Mutations in KLC have been linked to AD [105], and mutations in p150Glued and dynein are linked to motor neuron diseases such as amyotrophic lateral sclerosis (ALS) and distal spinal and bulbar muscular atrophy [106,107,108,109,110]. While the mechanisms of how these defects occur are unknown, perhaps problems in motor protein regulation or changes in motor protein levels could lead to degeneration of neurons and cell death.

Since the earliest known pathological feature of many degenerative diseases is the presence of axonal blockages or cytoplasmic accumulations within axonal processes [1,5,10,32,33], perhaps drugs that increase the function of motor protein activity could potentially be good candidates to alleviate axonal transport defects. While complications in the normal function of motors could arise by different mechanisms, all cause axonal transport defects. For example, problems could occur with cargo assembly or attachment of cargo to motors. Alternatively, mutations in the motor domain of motor proteins may alter motor activity or function. Problems could also arise due to improper attachment of the motor domain of motor proteins to MTs. Indeed, such defects in kinesin have been reported to cause HSP [104]. Additionally, in sick neurons, motor protein function could be over-activated for the transport of “rescue packages” to sites of injury or “signaling packages” from sites of injury. Therefore, drugs that inhibit or modulate the normal function of motor proteins could provide therapeutic relief. Alternatively, changes in tau phosphorylation could also affect the movement of motor proteins along MTs [111]. In this context, as discussed previously, drugs such as OKA that increase the levels of GSK-3β, which affect tau-MT interactions could be beneficial. Excess GSK-3β activity can also affect motor protein binding to vesicles during axonal transport ([9]. GSK-3β has also been shown to phosphorylate KLC, which is proposed to inactivate cargo binding [97]. Studies have also shown that GSK-3β can regulate dynein by phosphorylation of the DIC subunit [112]. Therefore, drugs like Peloruside A, which is thought to counteract GSK-3β activity, could augment kinesin and dynein-mediated motility within axons. However, it must be noted that since such drugs likely have additional affects, considering their broadly acting targets, studies to understand how to harvest their specific action on motor protein function might be required before they can be utilized as therapeutics.

One fundamental problem in using inhibitors of motor proteins is their lack of specificity as almost all of those currently available affect both kinesin and dynein motors. Nucleotide analogs serve as potent kinesin inhibitors; however, their lack of specificity makes them less efficient (*i.e.*, whether they target the ATPase domain, cargo attachment sites, or MT-binding sites). Furthermore, since it is likely that cooperation of both kinesin and dynein motors is necessary for proper transport along MTs *in vivo* [98,101], compounds that affect one motor will likely have secondary effects on the opposite motor for better or worse. Therefore, elucidating the mechanisms of how kinesin and dynein motors function cooperatively during bi-direction motility is important, as such studies will also benefit investigations on how to develop such marine drugs for specific motor actions as discussed below.

**Figure 3:**
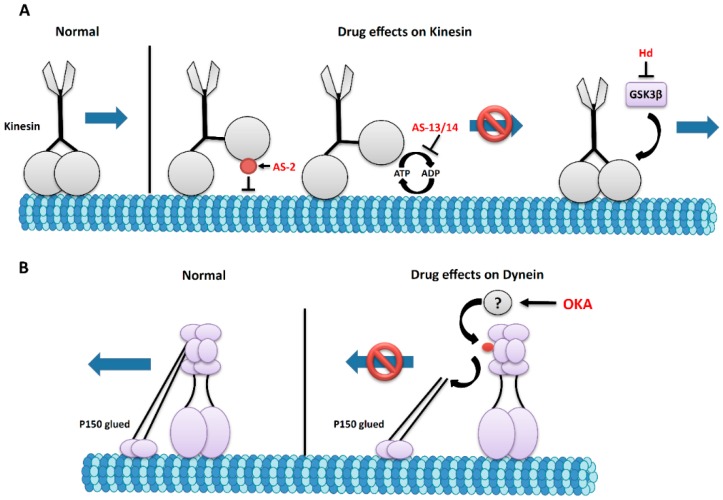
Proposed effects of marine drugs on the regulation of motor proteins. (**A**) The anterograde motor kinesin moves components from the cell body to the synapse (arrow). Adociasulfate 2 (AS-2) interacts with KHC and inhibits binding of KHC to MTs. Adociasulfates 13 and 14 (AS-13/14) inhibits the ATPase activity of KHC. Both of these mechanisms likely inhibit anterograde transport. Alternatively, GSK3β activity is associated with decreased attachment of vesicles to MTs [9] suggesting that the inhibitory effect of Hd on GSK3β could be useful in promoting anterograde transport; (**B**) The retrograde motor dynein transports cargo from the synapse to the cell body (arrow). OKA can increase the phosphorylation of dynein intermediate chain (DIC) which disrupts the association of the dynactin subunit p150glued with dynein; disrupting retrograde transport.

Adociasulfate-2 (AS-2), derived from the marine sponge *Haliclona* [113] specifically inhibits members of the kinesin superfamily (KHC, MPP1, MKLP1, RabK6, KIFC1, KIFC3, CENP-E, and Eg5) [114,115]. AS-2 is proposed to affect the motor domain of kinesins by inhibiting MT binding (Figure 3A) [114]. It is proposed that AS-2 forms extended aggregates that mimic the negatively charged MT surface thereby inhibiting kinesin activity [114]. However, AS-2 did not affect MT polymerization although the movement of kinesin along MT tracks was inhibited. *In vitro* studies showed that AS-2 binds to the MT-binding sites of the kinesin motor domain and that this binding can be reversed by increasing the concentration of MTs. In the absence of MTs, AS-2 stimulated the basal ATPase rate from 0.01/s to 0.06/s, indicating that AS-2 also affects motor activity.

Several other adociasulfates exist and function to inhibit kinesin motors. Adociasulfate-13 and -14, extracted from the sponge *Cladocroce aculeata,* compete with MTs for kinesin binding [116]. Both of these inhibit the ATPase activity of kinesin, which, in turn, blocks processive movement (Figure 3A). Inhibition of kinesin by these drugs is thought to be at a 1:1 interaction. AS-13 was shown to have the strongest inhibitory activity relative to all other adociasulfates [116].

Okadaic acid (OKA) is also proposed to increase the phosphorylation of dynein intermediate chain (DIC) [117]. Increased phosphorylation of DIC inhibits the ATPase activity of dynein. DIC is phosphorylated at the *N*-terminal region, where dynein binds p150Glued the subunit of dynactin [98]. Phosphorylation releases dynein from dynactin; severely impairing retrograde transport. Indeed, DIC phosphorylation was seen to be elevated in AD brain extracts, implying aberrant phosphorylation during AD [118]. Interestingly, work has shown a role for OKA in promoting AD-like neuropathology [119] by selectively inhibiting protein phosphatases PP1 and PP2A, which promotes hyper-phosphorylation of tau [49]. As a result, perhaps MTs can become destabilized causing motor proteins to dissociate from the MTs. Studies in primary cultures generated from rat cortical neurons revealed that OKA-treated neurons exhibit MT destabilization marked by accumulation of MTs around the soma and the proximal neurites [119]. After OKA treatment, the levels of DIC and p150Glued in these cultured neurons were reduced 40%–50% compared to controls; perhaps indicating the dissociation of dynein from dynactin (Figure 3B) disrupting motility on MTs [120]. Interestingly, inhibition of calpain cleavage partially rescued the MT accumulation seen with OKA treatment. The authors suggest that this is due to increased activity of dynein/dynactin which contains subunits that can normally be cleaved by calpain [119] further implicating OKA as a dynein inhibitor.

Marine drugs could also affect motor protein function by affecting particular kinases that are specific for the regulation of motor proteins. Interestingly, the previously mentioned compound hymenaldisine, which promotes MT stabilization by inhibiting GSK3β, could also prevent axonal blockages by modulating phosphorylation of kinesin and dynein due to the drug’s effect on GSK3β activity. Since increased GSK3β activity resulted in increased attachment of motors to membranes [9], drugs such as hymenaldisine which reduce GSK3b activity could stabilize motor attachment to vesicles restoring proper transport (Figure 3A).

C-Jun activating kinase (JNK) is another kinase that has been shown to affect the regulation of molecular motors during axonal transport. JNK phosphorylation of JNK-interacting protein 1 (JIP1) promotes the anterograde transport of JIP1, which associates with the phosphorylated form of amyloid precursor protein (APP) [121] and is important for APP transport within axons [122]. Conversely, activation of JNK3 (a neuronal specific JNK) resulted in increased phosphorylation of KHC and the detachment of kinesin from MTs [30]. Thus, marine drugs that affect the activity of JNKs could be important in regulating the activity of kinesin motors. Hioamide A and Plitidepsin are both cyclic depsipeptides found in marine organisms that cause increased activation of JNK [123]. As a result, these drugs could potentially be used to promote anterograde transport of JIP1-dependent cargoes such as APP-containing vesicles. However, such studies also indicate that these compounds cause increased neuronal apoptosis suggesting that they may also be toxic to cells. However, Plitidepsin is currently in a phase III trial under the brand name Aplidin for use as a potential cancer drug, suggesting its potential for therapeutics in humans [124]. Therefore, despite a drug being toxic, its therapeutic potential still warrants their use in humans. In addition, once the mechanisms of drug action are known, synthetic compounds can be generated to specifically enhance the benefits of a compound (specific targets such as motor protein function) and to decrease adverse effects (and broad effects) such that these drugs can then be effectively tailored to alleviate specific defects or to act on specific targets in humans.

## 6. The Autophagy-Lysosome Degradation Pathway: A Key to Neuronal Homeostasis and Survival

**Figure 4:**
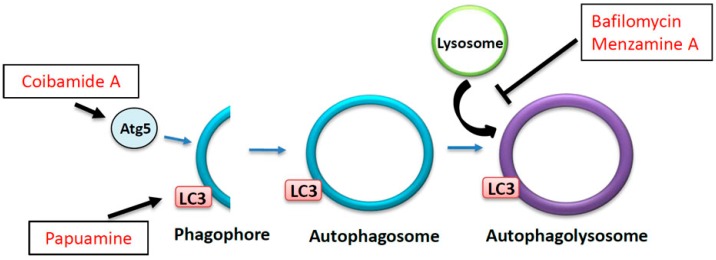
Proposed effects of marine drugs on the autophagy-lysosome pathway. The autophagy-lysosomal pathway is necessary for degradation of unneeded proteins, other non-essential cellular debris, and misfolded or aggregated proteins. A double-membrane phagophore forms around molecules to be degraded and ultimately forms the autophagosome. Fusion of autophagosomes with lysosomes forms the autophagolysosome where components are degraded. Marine drugs such as bafilomycins and menzamine A inhibit the fusion of lysosomes with autophagosomes and decrease the formation of autophagsomes. Alternatively, other marine drugs such as coibamide A and papuamine can increase upstream effectors of autophagy such as ATG5 and LC3, respectively, inducing the formation of autophagsomes.

Retrograde transport, dynein-mediated motility from the synapse to the cell body, is required to maintain cell homeostasis by degrading or recycling aging proteins and organelles from the nerve terminal to the cell body. The autophagy-lysosome pathway is one of the processes by which neurons degrade unneeded molecules. Autophagy has been shown to have a role in many physiological functions including organismal homeostasis, development, and tumor suppression. There are three major types of autophagy: macroautophagy, microautophagy, and chaperone-mediated autophagy. Macroautophagy is the main type of autophagy and is the process by which degradation occurs within specific membranous compartments called autophagolysosomes or autolysosomes (Figure 4). Membranes form around cytoplasmic compartments containing molecules and organelles for degradation. A double membrane invaginates to engulf molecules to be degraded. This process forms a semi-enclosed membrane called the phagophore. Enclosing of the double-membrane invagination forms the autophagosome. Maturation of autophagosomes occurs when it fuses with lysosomes to become an autolysosome where the degradation of cellular components occurs. A number of autophagy-related proteins (ATG1, ATG9, and ATG8) are required at various points along the pathway as autophagosomes form and ultimately mature into autolysosomes. Perturbations in the association of these complexes can result in aberrant degradation of molecules within the cell. ATG9 is a transmembrane protein that is conserved in mammals and is required for autophagosome formation [125,126]. Interestingly, ATG9 depends on the activities of ATG23 and ATG27 as well as the kinase ATG1 to bring ATG9 to or away from the forming autophagosome. Although not required for complete phagophore formation, these proteins are needed for efficient formation of the phagophore [127,128]. During autophagosome formation, an ATG5 complex, which is essential for formation of the autophagosome [129], temporarily binds to the outer membrane of the phagophore as it expands. Once the autophagosome is fully formed, ATG5 is released from the membrane [130]. ATG8 is necessary for maturation of autophagosomes [131], and also binds to membranes. Unlike ATG5, ATG8 remains on the autophagosome as it matures and is degraded together with other proteins [132]. Interestingly, recent studies have shown that the autophagy process in neurons depends on axonal transport [12,14,29]). Axonal transport of autophagy proteins and autophagosomes was also shown to be required for proper protein degradation [14].

In many neurodegenerative diseases, the autophagy-lysosome pathway is disrupted due to the sheer volume of misfolded, mutated, and/or aggregated proteins that need to be cleared from cells. The autophagy-lysosome pathway is unable to “keep up” with the misfolded protein load that is built up and becomes defective, causing the aggregation of proteins. Defects in autophagy have been reported in many diseases such as AD, PD, ALS, and HD [14,133,134], and all of these diseases exhibit pathologies defined by accumulation of aggregated disease proteins. One of the pathological hallmarks of AD is the formation of senile plaques containing amyloid-beta and intracellular NFTs containing aggregated hyper-phosphorylated tau in the brains of AD patients [48]. Patients with PD develop inclusions in the brain called Lewy bodies that contain aggregated proteins such as alpha-synuclein [135]. In HD patient brains, nuclear and cytoplasmic inclusions are observed which contain aggregations of expanded polyQ containing HTT [136]. In ALS, aggregation of super oxide dismutase (SOD) protein is observed in aggregated structures called bunia bodies [137]. Many of these aggregates also contain proteins in the autophagy-lysosome pathway and studies have recently shown a link between neurodegenerative diseases and problems in autophagy.

Interestingly, a mutation in sequestosome1 (SQSTM1), a marker for autophagy that binds cargoes, has been identified in patients with familial AD [138]. SQSTM1 has been shown to bind to dynein [139]. Perhaps SQSTM1 may link cargoes that need to be degraded to the retrograde motor. Additionally, reduction of the retrograde motor dynein has been shown to decrease the fusion of autophagosomes with lysosomes [14] indicating that the retrograde transport of autophagosomes within axons is important for their maturation. Furthermore, perturbations in axonal transport lead to defects in the autophagy pathway. Interestingly, mutant HTT containing expanded polyQ repeats reduced the retrograde movement of autophagosomes within axons causing defects in the fusion of autophagosomes with lysosomes [29]. Therefore, perhaps accumulation or aggregation of disease proteins within long narrow-caliber axons, due to defects in axonal transport may activate degradation pathways. Over time, these degradation pathways could become defective since the autophagy-lysosome system becomes overwhelmed with the extent of degradation that is necessary to achieve homeostasis. Therefore, while therapeutics aimed at repairing problems in axonal transport may be instrumental in efficient clearance of disease proteins, treatment strategies that stimulate degradation pathways could also be effective in clearing aggregates observed in many diseases.

## 7. The Effects of Marine Drugs on the Autophagy-Lysosome Pathway

Several marine drugs have been found to modulate the autophagy-lysosome pathway. As discussed previously, many neurodegenerative diseases are marked by reduced or inadequate levels of autophagic activity. Despite this, drugs that impair autophagy can give us insights into the mechanisms by which disease proteins impair the autophagy pathway. Bafilomycins, derived from marine *Streptomyces* sp., are widely used as inhibitors of autophagy (Figure 4). Addition of bafilomycin to cells causes increased levels of light chain 3 (LC3 or ATG8), a protein found in the membranes of autophagosomes [140]. Importantly, this increase in LC3 is caused by decreased fusion of autophagosomes and lysosomes rather than by simply lowering LC3 expression. Similarly, Manzamine A, derived from the marine sponge *Haliclona*, has been shown to negatively affect the fusion of autophagosomes to lysosomes and to suppress autophagy in pancreatic cells [141]. Interestingly, studies have shown that HD neurons exhibit reduced fusion between autophagosomes and lysosomes [14] and that small molecules aimed at increasing autophagy can reduce HD toxicity [142]. Perhaps bafilomycins are able to affect the maturation of autophagosomes to lysosomes by modulating the axonal transport of autophagosomes. Thus, these compounds can serve as useful tools for determining the precise mechanisms by which diseases like HD impair autophagy to aid in developing therapeutics.

Similarly, stimulating autophagosome formation in neurodegenerative disease states could speed up the process of degradation of misfolded or aggregated proteins as seen in the case of the small molecules used to promote autophagy [142]. Indeed, Coibamide A, which was isolated from a marine cyanobacterium, has been shown to induce autophagy in a manner that is dependent on ATG5 [143] (Figure 4). Another marine drug, Papuamine, isolated from *Haliclona*, has also been shown to induce autophagy by increasing the levels of LC3 [144] (Figure 4). Considering the effects of these marine compounds on autophagy, these drugs may be able to reduce the levels of misfolded and aggregated proteins that are seen in many neurodegenerative diseases. Indeed, protein aggregation in neurodegenerative diseases like AD and PD is congruent with upregulation of the unfolded-protein response (UPR) which has been shown to cause global decreases in protein production and cell death [145]. While further study is needed, perhaps increasing the levels of LC3 by Papuamine or similar compounds could increase the activity of the autophagy pathway, resulting in decreased levels of aggregated proteins in neurodegenerative diseases, thus restoring axonal transport and neuronal homeostasis.

## 8. Conclusions

Emerging links between impairment of the axonal transport pathway and the progression of neurodegenerative diseases suggest that transport defects occur early during disease progression. More and more studies are examining the importance of well-regulated axonal transport in the maintenance of homeostasis in neurons. In this review we have examined a number of marine compounds that cab affect the axonal transport system and have potential as therapeutics for neurodegeneration (Table 1). By taking advantage of compounds already found in marine environments, we cannot only enhance our knowledge of the mechanisms of how neurodegenerative disease initiates and propagates, but also evaluate how these compounds delay or even halt disease progression. Once a drug’s therapeutic potential is known, synthetic compounds can be generated that enhance benefits and decrease deleterious effects. Therefore, considering the adverse effects that these diseases have on our society, it is important that we examine all avenues for potential therapeutics aimed at curing neurodegenerative diseases, as no cures are currently available for any of these diseases. Thus, searching our oceans for potential therapeutic candidates is extremely beneficial and could even lead to identifying a cure for neurodegeneration.

## Figures and Tables

**Table 1:** Summary of marine drugs and their potential targets in the axonal transport pathway.

Axonal Transport Component	Compound	Source	Mode of Action	Reference
**Microtubles (MTs)**	Zamapnolide (ZMP)	*Fasicospongia rimosa*	Stabilizes MTs by enhancing tubulin assembly	[53]
	Dactyolide (DAC)	*Dactylospongia* sp.	Stabilizes MTs by enhancing tubulin assembly	[54,55]
	Hymenaldisine (Hd)	*Hymeniacidon aldis*, *Axinella verrucosa*, *Acanthella aurantiaca*	Destabilizes MTs by inhibiting phosphorylation of Tau	[56,57]
	Peloruside A	*Mycale hentscheli*	Stabilizes MTs by increasing levels of acetylated tubulin	[58,59]
	Discodermolide	*Discoderma dissolute*	Stabilizes MTs by targeting an additional binding site	[60,61,62]
	Okadaic acid (OKA)	*Halichondria okadai*	Destabilizes MTs. Inceases levels of phospho-tau and GSK-3β by inhibiting PP2A	[64]
	Dolastatin-10	*Dolabella auricularia*	Destabilize MTs by binding to the vinca alkaloid domain of tubulin	[63,65,66,67,68]
	Symplostatin	*Symploca hydnoides*	Destabilizes MTs similar to Dolastatin-10	[70]
	Halichondrin	*Halichondria okadai*	Destabilizes MTs by depolymerizing pre-existing MTs	[71,72,73]
	Cryptophycin	*Nostoc* sp.	Destabilize MTs by binding to the vinca alkaloid domain of tubulin and preventing GTP hydrolysis	[74]
**Molecular Motors**	Okadaic acid (OKA)	*Halichondria okadai*	Increases phosphorylation of DIC	[117]
	Adociasulfate-2 AS-2	*Haliclona*	Binds the kinesin motor domain and prevents kinesin movement	[113,114]
	Adociasulfate-13,14 (AS-13,14)	*Cladocroce aculeata*	Inhibits ATPase activity of kinesin	[115,116]
**Autophagy**	Bafilomycin	*Streptomyces*	Inhibits autophagy by preventing fusion of autophagosomes with lysosomes	[140]
	Manzamine A	*Haliclona*	Inhibits autophagy by preventing fusion of autophagosomes with lysosomes	[141]
	Coibamide A	Marine cyanobacterium	Induces autophagy in an Atg5 dependent manner	[143]
	Papuamine	*Haliclona*	Induces autophagy by increasing levels of LC3	[144]
